# Geographical Pattern of COVID-19-Related Outcomes over the Pandemic Period in France: A Nationwide Socio-Environmental Study

**DOI:** 10.3390/ijerph18041824

**Published:** 2021-02-13

**Authors:** Séverine Deguen, Wahida Kihal-Talantikite

**Affiliations:** 1 School of Public Health (EHESP), 35043 Rennes CEDEX, France; severine.deguen@ehesp.fr; 2 Department of Social Epidemiology, Institut Pierre Louis d’Epidémiologie et de Santé Publique (UMRS 1136), Sorbonne Universités, UPMC University Paris 06, INSERM, 75012 Paris, France; 3 Laboratoire Image Ville Environnement, LIVE UMR 7362 CNRS, University of Strasbourg, 67000 Strasbourg, France

**Keywords:** environmental inequalities, long-term exposure, air pollution, living condition, overcrowding housing, spatial disparities, COVID-19

## Abstract

Background: Several studies have investigated the implication of air pollution and some social determinants on COVID-19-related outcomes, but none of them assessed the implication of spatial repartition of the socio-environmental determinants on geographic variations of COVID-19 related outcomes. Understanding spatial heterogeneity in relation to the socio-environmental determinant and COVID-19-related outcomes is central to target interventions toward a vulnerable population. Objectives: To determine the spatial variability of COVID-19 related outcomes among the elderly in France at the department level. We also aimed to assess whether a geographic pattern of Covid-19 may be partially explained by spatial distribution of both long-term exposure to air pollution and deprived living conditions. Methods: This study considered four health events related to COVID-19 infection over the period of 18 March and 02 December 2020: (i) hospitalization, (ii) cases in intensive health care in the hospital, (iii) death in the hospital, and (iv) hospitalized patients recovered and returned back home. We used the percentage of household living in an overcrowding housing to characterize the living conditions and long-term exposure to NO_2_ to analyse the implication of air pollution. Using a spatial scan statistic approach, a Poisson cluster analysis method based on a likelihood ratio test and Monte Carlo replications was applied to identify high-risk clusters of a COVID-19-related outcome. Result: our results revealed that all the outcomes related to COVID-19 infection investigated were not randomly distributed in France with a statistically significant cluster of high risk located in Eastern France of the hospitalization, cases in the intensive health care at the hospital, death in the hospital, and recovered and returned back home compared to the rest of France (relative risk, RR = 1.28, *p*-value = 0.001, RR = 3.05, *p* = 0.001, RR = 2.94, *p* = 0.001, RR = 2.51, *p* = 0.001, respectively). After adjustments for socio-environmental determinants, the crude cluster shifts according to different scenarios suggested that both the overcrowding housing level and long-term exposure to largely NO_2_ explain the spatial distribution of COVID-19-related outcomes. Conclusions: Our findings suggest that the geographic pattern of COVID-19-related outcomes is largely explained by socio-spatial distribution of long-term exposure to NO_2_. However, to better understand spatial variations of COVID-19-related outcomes, it would be necessary to investigate and adjust it for other determinants. Thus, the current sanitary crisis reminds us of how unequal we all are in facing this disease.

## 1. Introduction

Over the last several months, most countries in the world have been impacted by the severe pandemic. In December 2019, in Wuhan (China), several pneumonia cases were observed [1]. These first patients suffered from an infection due to a novel coronavirus named SARS-CoV-2 for its similarity with the epidemic. Severe Acute Respiratory Syndrome (SARS) occurred between 2002 and 2003. Patients affected by SARS-CoV-2 infection could experience serious complications, including pulmonary oedema, severe pneumonia, and acute respiratory stress syndrome, which, in several cases, could turn into death. Several risk factors of most severe symptoms, which may turn to intensive care recovery, were identified as age and existence of comorbidities such as respiratory diseases [2,3,4]. The outbreak of the novel coronavirus (defined with the acronym COVID-19) is an ongoing global epidemic that was declared as a pandemic in March 2020 by the World Health Organization [5].

Italy was the first country in Europe to be affected by the Covid-19 epidemic [6], which was followed rapidly by other European countries such as Spain and France. In France, the epidemic started in February with three main clusters located in Grand-Est, Britany, and Haut de France regions. Rapidly, the outbreak spread over the national territory, which lead the French government [7,8] to declare the lockdown of the French population for two weeks on 16 March, 2020. The lockdown was extended thereafter for two months. As reported in Italy, the ongoing epidemic revealed strong geographical differences in the spread of infection with a majority of cases concentrated in Northeast France including Ile-de-France, Haut-de-France, and Grand-Est regions. This unequal spatial distribution of the infection spread, combined with the healthcare capacity of each region, lead the government to classify the French region in two categories (green and red regions) guiding the un-containment plan started on 11 May 2020.

While many recent studies focused on individuals ‘risk factors of COVID-19 infection, which constitute the vulnerable population, few analysed the possible role of characteristics measured at the residential place, including environmental nuisances and socioeconomic deprivation. During the last few months, a couple of studies investigated the relationship between air pollution and COVID-19 morbidity as well as mortality in different countries [6,9,10,11,12,13,14].

A systematic review was recently published to summarize the scientific evidence on the role of air pollution (PM and Nitrogen dioxide, NO_2_) in COVID-19 spread and lethality. The authors revealed that major findings are consistent, highlighting the important contribution of PM_2.5_ and NO_2_ as triggering COVID-19 spread and lethality [15]. More studies are needed to strengthen scientific evidence and support firm conclusions. The systematic review describe studies investigating the relationship between air pollution and COVID-19 morbidity and mortality in different countries [6,9,10,11,12,13,14]. While some studies investigated the role of short-term exposure to air pollution [11,12], others explored the role of chronic-exposure to air pollution [6,9,10].

The authors measured long-term exposure from different periods including two months prior to the outbreak of COVID-19 in Europe [9], four years (2016–2019) in Italy [6] while, in US, they considered a longer period from 2000 to 2016 [10,13]. More precisely, the Italian study [6] revealed that the higher level of air pollution estimated in the north part of Italy may partially explain the regional differences regarding the COVID-19 infection between the north and south part of the country. Such a link was already reported during the 2003 SARS outbreak in China, where Cui et al., 2003, revealed that infected people who lived in regions with a high air pollution index were twice as likely to die as those living in regions with a low air pollution index [16]. Overall, these studies suggest that the chronic exposure to higher levels of air pollution may contribute to COVID-19 severity or to health events related to COVID-19.

On the other hand, several studies suggest the association of poor housing conditions with COVID-19 incidence and mortality. The U.S. study revealed that counties with a higher percentage of households with poor housing had a higher incidence of, and mortality associated with, COVID-19 [17]. Thus, the consideration of long-term exposure to air pollution and deprived living condition as an environmental hazard may improve our understanding of the COVID-19 pandemic. Therefore, the question addressed here is: are the rate of health events related to COVID-19 higher among peoples living in a more polluted and more deprived living condition? In other words, is there a socio-environmental disproportionate distribution of COVID-19-related outcomes? In this context, the aim of the present study is to investigate whether a geographic pattern of Covid-19 may be explained by spatial distribution of both long-term exposure to air pollution and a deprived living condition by assessing the spatial implication of neighbourhood characteristics on a spatial repartition of a Covid-19-related outcome.

## 2. Materials and Methods

### 2.1. Study Area

The study area is the national territory of the France metropolitan, which host about 64 million inhabitants (INSEE, 2020). France is subdivided into 95 departments with a mean population of about 680,000 inhabitants (varying from a minimum of 76,000 inhabitants in Lozère and more than 2 million inhabitants in Nord, Bouches-du-Rhône, and Nord departments).

### 2.2. Health Data

This study considered four health events related to a COVID-19 infection. All indicators were estimated at the French department scale. We used health data collected in the hospital available from the French government website [18]. The French Public Health Agency (Santé Publique France) collected all health data of confirmed cases of COVID-19 from all health care systems in order to monitor the pandemic. All anonymized databases at the department level were available on open access from the health minister website.

Four health indicators were considered, over the period of 18 March and 02 December 2020.

i. the total number of hospitalized persons due to COVID-19 infection,
ii. the total number of severe COVID-19 cases in the intensive health care in the hospital,
iii. the total number of deaths at the hospital caused by COVID-19 infection, and
iv. the total number of hospitalized patients recovered and returned back home.

All number cases as well as the number of deaths have been divided by the department number of inhabitants to standardize the health indicators by population size. The number of inhabitants per department are available from the National Institute of Statistics and Economic Studies (INSEE website [19]).

### 2.3. Air Pollution Data

Several recent epidemiological studies and scientific evidence suggest the role of air pollution in COVID-19 spread and lethality [15] including NO_2_ and PM. The Nitrogen dioxide (NO_2_) is a good indicator for traffic-related air pollution and correlated with other traffic-related air pollution: particulate matter. In addition, the modelled NO_2_ presents more spatial variations than other modelled outdoor pollutants [20]. Today, NO_2_ may be considered a good proxy indicator of outdoor air pollution. Therefore, Nitrogen dioxide (NO_2_) was the pollutant included in this study. This pollutant was known to be a good marker of the pollution due to road traffic. It was chosen because of the largest spatial variabilities observed compared to particle matters. The dataset of annual mean of NO_2_ for each monitoring station was taken from 12 Associations for Surveillance of Air Quality (AASQA) through the National Association for the Surveillance of Air Pollution (ATMO France) [21] and from the European Environmental Agency, accessed on 22/05/2020, EEA, 2020).

We estimated the average of the annual mean of NO_2_ between 2014 and 2018 for all monitoring stations (Scenario 0) for background and traffic monitoring stations (Scenario 1) and for background monitoring stations only (Scenario 2) located within each department in France. Then, we obtained tree measures of NO_2_ exposure per department, which reflect the long-term NO_2_ exposure of the population.

### 2.4. Neighbourhood Deprivation Context

To characterize the deprived condition level, in this study, we used indicators to characterize the living conditions during the containment: the percentage of household living in an overcrowding housing. Data are collected by the National Institute of Statistics and Economic Studies (INSEE) and available on their website [22].

### 2.5. Descriptive Analysis

First, the Pearson correlation coefficient “r” was produced in order to quantify the intensity of the relation between each health event and the deprived living condition, as well as the long-term NO_2_ exposure separately.

Second, we completed the descriptive analysis by implementing a simple linear regression to give an order of magnitude of the health rate increase according to the increase of the covariates (living condition and long-term exposure to NO_2_).

Finally, in order to explore the existence of a potential interaction between the socioeconomic variable and NO_2_ exposure, we stratified our analysis in two groups based on the median of the distribution of the percentage of household living in an overcrowding housing. Interactions were formally tested by introducing an interaction term in the linear regression to produce the *p*-value and conclude the statistical significance of the modifier effect of overcrowding in relation to air pollution and health events related to a COVID-19 infection.

### 2.6. Spatial Analysis

To investigate the geographic pattern of the incidence of a Covid-19 related-outcome at the department level in France, we used the most appropriate spatial scan statistic approach [23] implemented in the SaTScan software [24]. This approach is used in an increasing number of applications in the field of spatial epidemiology and public health [25,26,27,28,29]. In our study, its allows the statistical and significant investigation of the presence of a high-risk Covid-19-related outcome among elderly people during pandemic periods and their spatial approximate location [30,31,32].

### 2.7. Methodological Approach

In this approach, the null hypothesis (H_0_) tested is that the risk of a Covid-19 health-related outcome is the same throughout the study area. In other words, the expected incidence of a COVID-19-related outcome would be randomly distributed in space [28,33].

The alternative hypothesis (H_1_) is that there is an elevated risk of incidence of a COVID-19-related outcome within the cluster in comparison with census blocks outside the cluster.

The Poisson probability model was chosen as a cluster analysis method to detect the presence of cluster of high risk of a COVID-19-related outcome. More precisely, in our approach, we used the Poisson-based model where the number of COVID-19 health events in a geographical area is Poisson-distributed.

The procedure works as follows: a circle or window of a variable radius (from 0% up to 50% of the population size [34]) is placed through an iterative process on one centroid of the department and moves across the whole study area, to compare the incidence of a COVID-19-related outcome in the window with incidence expected under a random distribution.

**Step 1: Estimate the relative risk (RR) within each circle based on a Poisson model**

We, therefore, computed a relative risk (RR) in each department weighted by the population size living in each department. This RR represents the expected risk of a COVID-19-related outcome within an area or window divided by the expected risk outside of the department or window.

We conducted separate analysis for each incidence of a COVID-19 related outcome (i.e., hospitalization, death, severe form, return back home). This is the estimated risk within the cluster divided by the estimated risk outside the cluster. It is calculated as the observed divide by the expectation within the cluster, which is divided by the observation expected outside the cluster.

The mathematical notation is described below with Equation (1).

(1) $$\mathrm{RR}=\frac{c/E\left[c\right]}{\left(C-c\right)/\left(E\left[C\right]-E\left[c\right]\right)}=\frac{c/E\left[c\right]}{\left(C-c\right)/\left(C-E\left[c\right]\right)}$$

where:

▪ c is the number of observed cases within the cluster and
▪ C is the total number of cases in the data set.
▪ Note that since the analysis is conditioned on the total number of cases observed, E[C] = C.

**Step 2: Identification of the most likelihood cluster**

An increasing number of circles with different sets of departments within them are created. Thus, each circle is a possible candidate for a cluster with the likelihood estimate. The scan statistic approach is likelihood based. The most likely cluster can be selected and tested for statistical significance.

Under the Poisson assumption, the likelihood function is described by Equation (2).

(2) $${\left(\frac{c}{E\left[c\right]}\right)}^{c}{\left(\frac{C-c}{C-E\left[c\right]}\right)}^{C-c}I()$$

where:

▪ C is the total number of cases,
▪ c is the observed number of cases within the window, and
▪ E[c] is the covariate adjusted and expected number of cases within the window under the null-hypothesis.
▪ Note that, since the analysis is conditioned on the total number of cases observed, C-E[c] is the expected number of cases outside the window.
▪ I() is equal to 1 when the window has more cases than expected under the null-hypothesis, and 0 otherwise. Since this analysis is only interested in detecting clusters with higher than expected rates, I() was equal to 1.

In the first crude model, which is the unadjusted analysis, E[c] is equal to an expected number of cases within the window under the null-hypothesis. In the second and third adjusted model, E[c] is a covariate adjusted and expected number of cases.

The likelihood function is maximized over all window locations and sizes, and the one with the maximum likelihood constitutes the most likely cluster. The identification of the most likely clusters is based on a likelihood ratio test [34] with an associated *p*-value obtained using Monte Carlo replications [33]. We considered a 0.05 level of significance. Mapping of the clusters was carried out using ArcGIS [35].

### 2.8. Methodological Strategy

If we detect a significant cluster of high-risk using this method, the next step will be to explore whether the significant cluster can be explained by suspected risk factors. Thus, spatial analyses were performed in four stages (step-by-step).

i. Crude analysis (unadjusted) to identify and spatially localize the most likely cluster of high incidence of a COVID-19 related outcome.
ii. Adjusted analysis for a living deprivation condition.
iii. Adjusted analysis for long-term exposure to NO_2_.
iv. Adjusted analysis for both the deprivation context and long-term exposure to NO_2_.

The models were adjusted on one or more co-variables, and, according to the Kulldorff studies [34], several criteria were used to reject, or not, the H_0_ hypothesis according to the cluster’s localization and statistical significance, as well as the likelihood ratio value of each model (for more detail, see References [25,26,27,36]).

## 3. Results

### 3.1. Spatial Description

**Figure 1:**
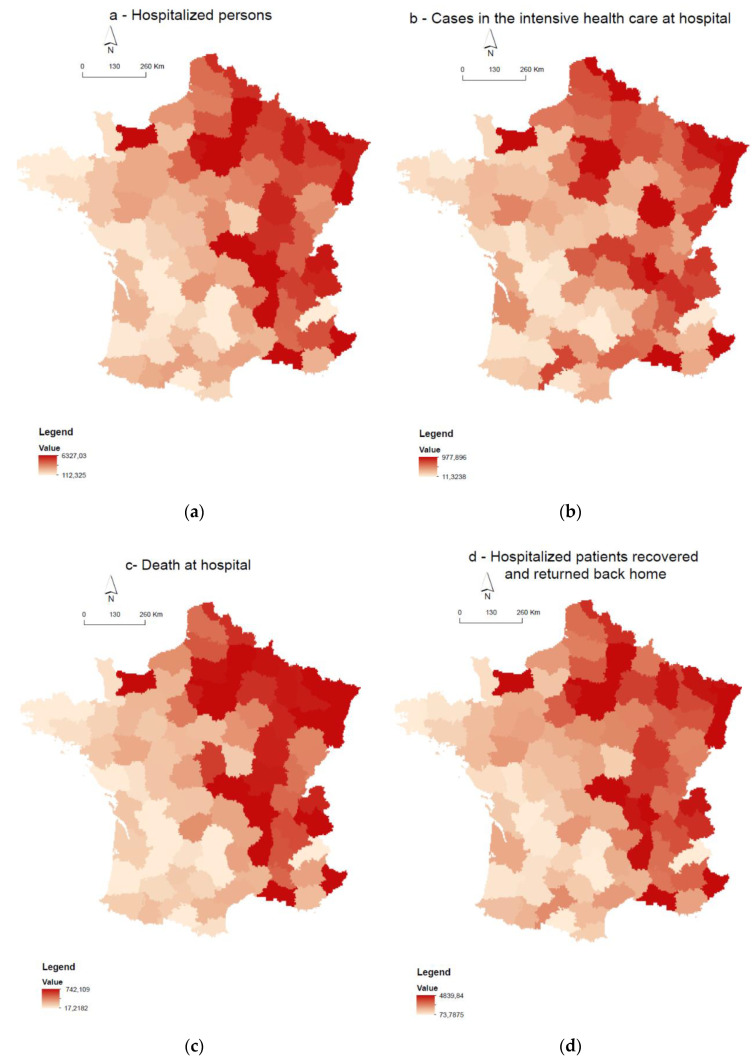
Spatial distribution on the French department scale. (**a**) Rate of death in the hospital for 100,000 inhabitants, (**b**) rate of hospitalized persons for 100,000 inhabitants, (**c**) cases hospitalized in intensive health care for 100,000 inhabitants, and (**d**) rate of hospitalized patients recovered and returned back home for 100,000 inhabitants.

**Figure 2:**
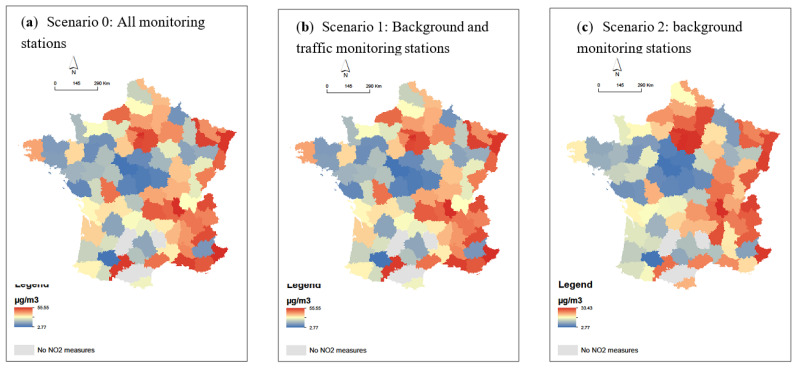
Spatial distribution on the French department scale of the annual mean of NO_2_ between 2014 and 2018 (**a**) for all monitoring stations (Scenario 0), (**b**) for background and traffic monitoring stations (Scenario 1), and (**c**) for background monitoring stations only (Scenario 2).

**Figure 3:**
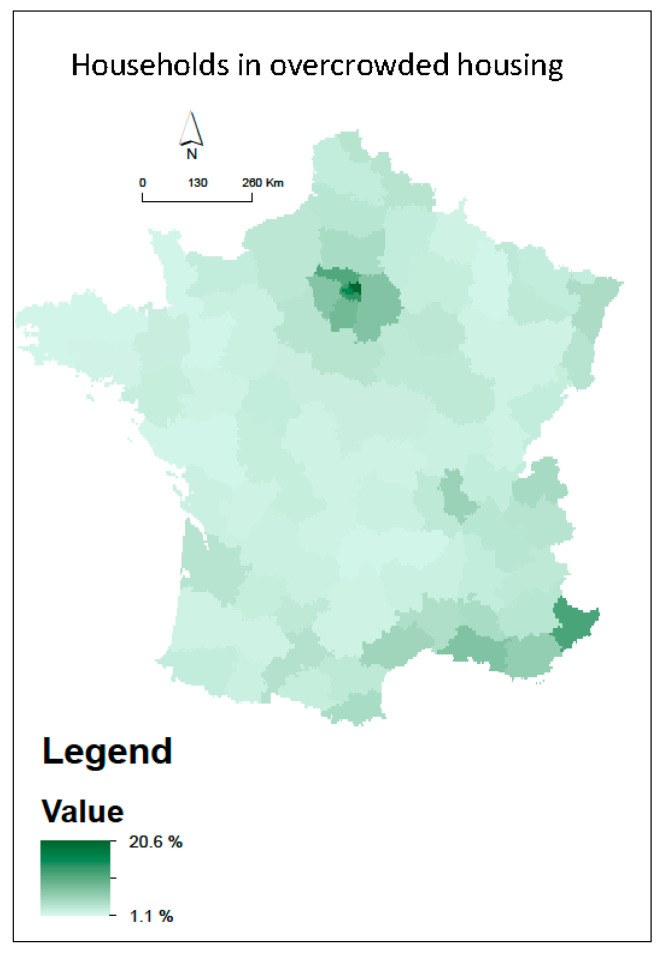
Spatial distribution on the French department scale of the percentage of households living in overcrowded housing.

Figure 1a–d presents the spatial distribution of the four health events related to COVID-19 infection, which reveal a strong spatial pattern with higher health events counted in Northeast France. The annual mean of NO_2_ is higher in the north and east departments of France whatever the scenario (Figure 2). As expected, from the background monitoring stations (scenario 2), less variabilities of NO_2_ are observed (Figure 2c). Spatial clusters of a high percentage of households living in overcrowded housing are visible in the Ile de France region and in the departments near the Mediterranean Sea (Figure 3).

### 3.2. Descriptive Data

**Figure 4:**
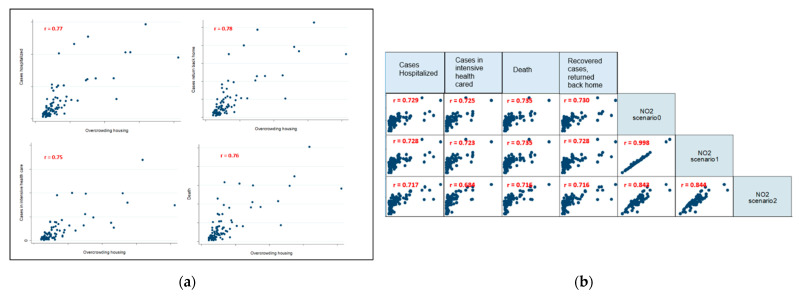
Correlation between health events that are COVID-19-related and deprived living conditions (**a**), long-term NO_2_ indicators (**b**).

The fours graphics in Figure 4a revealed significant linear associations between the percentage of households living in overcrowded housing and each of the four health indicators: the correlation coefficients vary between 0.75 and 0.78. All are statistically significant. In the graphics of Figure 4b, similar results were found with long-term NO_2_ exposure regardless of the monitoring stations included in the calculation of the air pollution indicators (Figure 4b). The NO_2_ exposure is higher over the long-term, and the rate of health events is higher. The correlation coefficients vary between 0.68 and 0.735.

Table 1 presents the association between the overcrowding housing and COVID-19 health events. All measures of associations are highly significant (*p* < 0.0001), between overcrowded housing and four COVID-19 related outcomes. The regression coefficients estimated by a linear varied from 71.8 into 439.4 for cases hospitalized and cases in intensive healthcare, respectively.

Table 2 presents the association between long-term NO_2_ exposure and health events stratified, according to tertile distribution of the percentage of overcrowded housing. All measures of associations are highly significant among the department with a high percentage of households living in overcrowded housing. These findings are in favour of the existence of an interaction between a deprived living condition and long-term NO_2_ exposure on intensive healthcare risk, as confirmed by the interaction tests (*p* = 0.008).

## 4. Spatial Distribution

**Figure 5:**
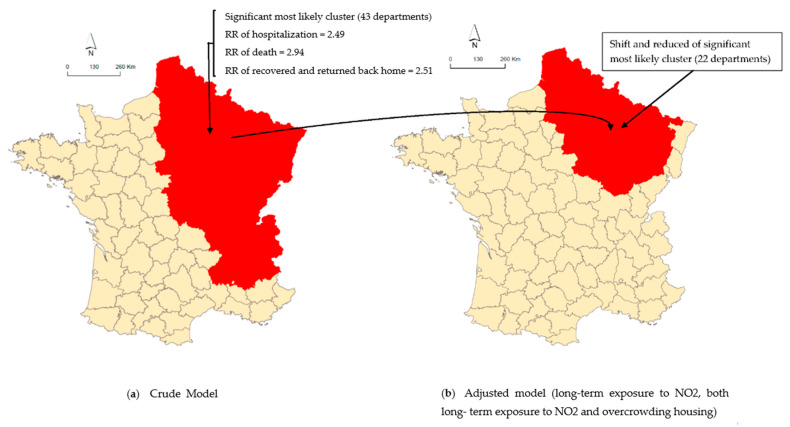
Mapping of a spatial display of incidence of COVID-19 related outcome (Hospitalization death in the hospital, recovered, and returned back home): (**a**) crude model, (**b**) adjusted model on long-term exposure to NO_2_, and both long-term exposure to NO_2_ and overcrowded housing.

Figure 5 details the department containing the most likely clusters of high risk of a COVID-related health incidence (hospitalized, intensive health care, death, recovered cases, returned back home), their spatial location, and the spatial shift of centroid from an unadjusted cluster to a covariate-adjusted cluster. Table 3 presents the most likely clusters, the number of departments, the radius of the circle of the cluster, and the relative risk (RR, the ratio of the observed-to-expected number of new patients in each department estimated by SaTScan) for each cluster.

### Unadjusted Analysis

Figure 5a revealed the location of the most likely cluster. Regardless of the COVID-19-related outcome, the most likely cluster had a statistically significant high risk of a COVID-19-related outcome incidence. More precisely, the result reveled that the risk of a COVID-19 hospitalized incidence was 2.49 greater in the eastern part than the rest of France (*p*-value = 0.001, Table 3). The spatial analysis of COVID-19-related death, which was recovered and returned back home revealed the same most likely clusters located in Northeast France and composed of 43 departments. The clusters had a risk of death and, of those recovered and returned back home, 2.94 and 2.51 (respectively) were greater than the rest of France. A small cluster of intensive healthcare in the hospital was also identified in Northeast France (*p*-value = 0.039). The cluster composed of six departments had a risk of intensive healthcare in a hospital of 3.05 greater than the rest of France (*p*-value = 0.0001, Table 3).

Adjusted scan statistical analysis is detailed below according to the variables for which the model was adjusted.

After adjustment for the percentage of households living in overcrowded housing. The most likely cluster remained statistically significant and located in the same zone. However, the likelihood ratio decreasing from 22,089 to 11,756 indicates that the spatial distribution of overcrowded housing only slightly explains the spatial distribution of a COVID-19-related outcome (data not shown).

After adjustment for long-term exposure to NO_2_ (Figure 5b), the cluster was reduced, the centroid of the cluster was shifted to Northeast France and the likelihood ratio decreased from 22,089 to 3074 (Table 3). It indicates that the spatial distribution of long-term exposure to NO_2_ partially explained the excess risk of COVID hospitalization observed in the unadjusted analysis.

The cluster of excess risk of death identified in crude analysis in Eastern France was also reduced from 43 departments to 22 departments, after an adjustment for long-term exposure to NO_2_ and the likelihood ratio largely decreased from 4642.46 to 855.92. In the same pattern, the cluster of excess risk of recovered values and those returned back home identified in crude analysis in Eastern France was also reduced from 43 to 25 departments and the likelihood ratio largely decreased from 15,673.73 to 2247.95 (Table 3).

In addition, the cluster of excess risk of intensive health care in a hospital identified in crude analysis in Eastern France was also reduced: the number of departments decreased from 6 to 4, after adjustment for long-term exposure to NO_2_. The likelihood ratio decreased from 3906.31 to 825.26 (Table 3).

After adjustment for long-term exposure to NO_2_ and percentage of households living in overcrowded housing (Figure 5b)—The most likely significant cluster shifted in Northeastern France (RR = 1.32) with a relatively larger decrease in the likelihood ratio from 22,089 to 2189 (Table 3). These results indicated that long-term exposure to NO_2_ and percentage of households living in overcrowded housing explained a great part of the excess risk of COVID-19 hospitalization observed in the unadjusted analysis.

The cluster of excess risk of death identified in crude analysis in East France was also reduced to 22 departments, after an adjustment for long-term exposure to NO_2_ and a deprived conditional level. The Llr value largely decreased from 4642.46 to 601.95. Similarly, the cluster of excess risk of recovered and returned back home identified in crude analysis in Eastern France was also reduced to 22 departments and the Llr largely decreased from 15,673.73 to 1605.65 (Table 3).

In addition, the cluster of excess risk of intensive healthcare in a hospital identified in crude analysis in Eastern France was also reduced to a cluster of 4 departments. The likelihood ratio largely decreased from 3906.31 to 555.34 (Table 3).

Taking in to account the long-term exposure to NO_2_ and the percentage of households living in overcrowded housing reduced the LLr to a larger degree than long-term exposure to NO_2_ alone. These variables also explain a large amount of the excess of a COVID-19-related outcome.

Our results indicated that the excess risk of different COVID-19-related outcomes among a population aged over 60 years old observed in the unadjusted analysis was explained in a great part, but not entirely, by long-term exposure to NO_2_ and the percentage of households living in overcrowded housing.

## 5. Discussion

Our nationwide study in France revealed a significant correlation between long-term exposure to NO_2_ and incidence of a COVID-19-related outcome. To our knowledge, such a work, exploring spatial implication of long-term exposure to NO_2_ on geographical variations of a COVID-19 related outcome, has never been performed. It is one reason why it is difficult to compare our findings with those of others.

Our study revealed that all the outcomes related to COVID-19 infection investigated (including: hospitalization, death at hospital, recovered and returned back home, intensive health care in a hospital) were not randomly distributed in France. The increased COVID-19-related outcome incidence in Eastern France was statistically significant. The spatial distribution of both long-term exposure to NO_2_ and overcrowded housing may be taken into account to fully interpret the spatial distribution of incidence of COVID-19-related outcomes.

These findings are in line with recent published studies investigating the role of air pollution in COVID-19 infection, including mortality and morbidity outcomes.

More precisely, our results are consistent with studies carried out in European countries as well as in US and China, which suggest that people living in a polluted area are more predisposed to develop severe COVID-19-related events [6,9,10]. In Europe, Ogen et al., 2020, revealed that 78% of death related to COVID-19 identified in Europe are mainly concentrated in five polluted regions located in North Italy and Central Spain. More precisely, the authors found that 83% of all fatalities occurred in the European region where the maximum NO_2_ concentrations was above 100 µmol/m^2^ [9]. In the United States, Wu et al. suggested that even a small increase in long-term exposure to PM_2.5_ increase the COVID-19 death rate: 1µg/m^3^ in PM_2.5_ is associated with an 8% increase in the COVID-19 death rate (95CI [2%, 15%]) [10]. All these results enrich the debate of the potential effects of chronic exposure to air pollution on the COVID-19 severity.

Therefore, we may hypothesize that chronic exposure to a high level of air pollution and living condition (approximated here by the percentage of households living in overcrowded housing) could contribute to spatial, disproportionate, severe forms in COVID-19-related outcomes observed following the different pathways.

(i) the first one acts indirectly by increasing the risk of cardiopulmonary and respiratory diseases [37,38] as well as the hypertension and the diabetes [39], which were identified as one main comorbidity risk factor of COVID-19,
(ii) the second one has a more direct effect by increasing susceptibility of people to COVID-19 infection.

In addition, our finding suggests that both an overcrowded housing level and chronic exposure to NO_2_ could largely explain the spatial distribution of COVID-19-related observations in a French metropolitan area.

Our finding suggests an unequal impact of COVID-19 including a death, COVID-19 hospitalization, recovered and returned back home, and intensive healthcare in a hospital. We argue that this unequal geographic pattern is due to a complex interaction between the outdoor and indoor condition: air pollution and housing condition. Thus, beyond the air pollution effect, the living condition may contribute to an unequal impact of COVID-19.

As suggested by Patel et al., 2020: “To date, policymakers have targeted people with multiple comorbidities after identifying them as the most vulnerable. However, this medical model of disease risks ignoring social factors, which can increase exposure to and mortality from coronavirus disease 2019 (COVID-19)” [40].

Based on a conceptual model of Singer [41], and the model of the main determinants of health described by Dahlgren and Whitehead [42], Bambra et al. proposed a theoretical framework of “the syndemic of COVID-19, non-communicable diseases (NCDs) and the social determinants of health”. His framework aims to explain how inequalities in COVID-19 are related to existing inequalities in chronic diseases and the social determinants of health” [43]. Thus, the authors argue that, for the most disadvantaged communities, COVID-19 is experienced as a syndemic—a co-occurring, synergistic pandemic that interacts with and exacerbates their existing NCDs and social conditions [43].

Among the living condition, housing characteristics play an important role in unequal impact of COVID-19 by following some pathways:

(i) Some studies suggest that people in lower income households are more likely experience overcrowding and live in overcrowded conditions. Therefore, deprived living conditions may constitute itself as an additional risk factor of the known underlying clinical risk factors that increase the severity and mortality of COVID-19 (including cardiovascular disease, obesity, diabetes, and hypertension [44]). It suggests that people living in deprived conditions have an increased susceptibility to COVID-19 mortality.
(ii) The overcrowding combined with poor quality of housing conditions may increase the vulnerability of people for COVID-19 and the severity of its consequences. These deprived living conditions including damp housing and overcrowding may induce some health outcome respiratory disorders, such as asthma and other viral infections.

Our hypothesis highlights the complexity of the mechanisms, which link chronic exposure to NO_2_ and living conditions to COVID-19-related outcomes. It suggests that the combination of outdoor and indoor conditions may interact to explain the geographical pattern of COVID-19.

Lastly, our findings showed that it remained a significant cluster of excess risk of COVID-19-related outcomes, not entirely explained by spatial distribution of age, NO_2_, and overcrowded housing. This observed cluster cannot be explained by healthcare access. In France, the access to diagnosis and treatment should not be limited by socio-economic status. Medical and hospital costs for patients with COVID-19-related outcomes are completely covered (100%), and the reimbursement is regulated by uniform rates regardless of whether the patient is treated in the public or private facility.

This observed cluster may be partly explained by the population health status such as the pre-existing chronic diseases related to the COVID-19-related outcome known as a risk factor [45].

Interpretation of our findings must consider weaknesses that could affect the strength of the associations, yield limitations in comparison with other studies, or impede the formulation of accurate conclusions.

- First, we performed an ecological study with data available at the French department level. Therefore, our results should be interpreted only in this design context and should not be interpreted at the individual level.
- Second, our approach based on ecological data, has several limitations. One is the non-inclusion of gender and presence of pre-existing and background diseases and comorbidities in the analysis, which is known to be risk factors for COVID-19-related outcomes. Focusing in further studies on larger risk factors is recommended, and might produce clearer results to explain the cluster of the excess risk.
- Third, to characterize the chronic exposure to NO_2_, as recent studies investigated this issue, we used mean values over five years (2014–2018) from all monitoring stations located in each French department, including several background, traffic, and industrial stations. The mean values from each station may vary according to the type: background, traffic, and industrial station, and to the area: rural, urban, and sub-urban. In our study, we used all data available at the French department level and carried out sensitivity analysis through three scenarios. However, in our study, using data from a different type of monitoring stations may misclassify the level of exposure of several French departments. At this time, these were the only available data for the study period, which covered all departments of France. However, the results revealed that the measure of associations as well as the statistical significance did not vary as much, according to the scenario used (data not shown). Next, studies need to assess the impact of air pollution on COVID-19 outcomes using modelled measures of NO_2_ exposure at a finer spatial scale.

## 6. Conclusions

Our study quantified the unequal spatial distribution of the COVID-19 related outcome. The clustering analysis confirmed the higher risk of COVID-19-related outcome located in Eastern France. In addition, our findings suggest that the geographic pattern of a COVID-19-related outcome is largely explained by socio-spatial distribution of long-term exposure to NO_2_. However, to better understand spatial variations of a COVID-19-related outcome, it would be necessary to research and adjust for other determinants.

Thus, the current sanitary crisis reminds us how unequal we all are in facing this disease. Building a healthy environment for all, and especially for the most vulnerable population, is a crucial issue if we want to design and implement measures for a greener and more equitable territory.

## Figures and Tables

**Table 1:** Coefficient estimate of linear regression adjusted on the proportion of population aged over 60 years old.

Health Event	Overcrowded Housing *	
	Beta-Coefficient	*p*-Value
Cases hospitalized	439.4	<0.0001
Cases in intensive healthcare	77.5	<0.0001
Death	71.8	<0.0001
Recovered cases, returned back home	330.6	<0.0001

* Linear regression adjusted on the proportion of population aged over 60 years old.

**Table 2:** Association between long-term NO_2_ exposure (scenario 2) and COVID-19-related outcomes according to the tertile distribution of the percentage of overcrowded housing.

Health Event	Household Living in Overcrowded Housing *						Interaction Test *p*-Value **
	Tertile 1		Tertile 2		Tertile 3		
	Beta-Coefficient	*p*-Value	Beta-Coefficient	*p*-Value	Beta-Coefficient	*p*-Value	
Cases hospitalized	39.6	0.078	61.3	0.032	183.7	<0.0001	0.121
Cases in intensive healthcare	4.79	0.0814	10.1	0.018	35.5	<0.0001	0.008
Death	7.04	0.090	11.4	0.06	32.2	<0.0001	0.094
Recovered cases, returned back home	24.0	0.119	41.7	0.031	136.9	0.002	0.312

* Linear regression adjusted on the proportion of population aged over 60 years old. ** *p* value of the interaction test to evaluate statistical significance of the modifier effect of overcrowding in relation to air pollution and health events related to a COVID-19 infection.

**Table 3:** Summary statistics of the most likely clusters of COVID-19-related outcomes and spatial relocation resulting from the adjustment analysis.

Analysis	Cluster Radius ^b^	Number of Departments ^c^	No. of Observed Cases ^d^	No of Expected Cases ^e^	RR	LLr	Shift ^f^	*p*-Value ^g^
Unadjusted No adjustment (Crude) ^a^								
Hospitalization	536.19	43	166,571	116,576.69	2.49	22,089.95		0.001
Intensive healthcare at hospital	80.27	6	10,549	4267.91	3.05	3906.31		0.001
Death at hospital	536.19	43	27,241	18,229.86	2.94	4642.46		0.001
Recovered and returned back home	536.19	43	117,321	82,005.68	2.51	15,673.73		0.001
Adjusted analysis for long-term exposure to NO_2_								
Hospitalization	285.12	22	99,987	81,646.92	1.39	3074.93	Yes	0.001
Intensive healthcare at hospital	16.57	4	8461	5501.48	1.70	825.26	Yes	0.001
Death at hospital	285.12	22	16,731	12,883.78	1.55	855.92	Yes	0.001
Recovered and returned back home	317.61	25	77,346	63,957.82	1.40	2247.95	Yes	0.001
Adjusted analysis for long-term exposure to NO_2_ and deprived level (Occupation)								
Hospitalization	321.93	37	131,368	115,393.32	1.32	2189.38	Yes	
Intensive healthcare at hospital	16.57	4	8461	5982.79	1.54	555.34	Yes	0.001
Death at hospital	285.12	22	16,731	266.4	1.44	601.95	Yes	0.001
Recovered and returned back home	285.12	22	71,241	60,053.16	1.33	1605.65	Yes	0.001

RR: relative risk. LLr: log likelihood ratio. ^a^ Unadjusted analysis, to identify and localize the most likely cluster(s) of high risk of COVID-19-related outcomes. ^b^ Cluster radius is the radius of the spatial circle. ^c^ Number of Department: is the number of departments composed of the most likely cluster. ^d^ No. of observed cases: the number of the observed cases within the most likelihood cluster. ^e^ No. of expected cases: the number of the expected cases within the most likely cluster. ^f^ Shift: if the cluster is in the same location of the crude, the most likely cluster (in unadjusted analysis). ^g^ *p* value: the statistically significant one of the most likely cluster.

## Data Availability

Avalaibility of socioeconomic data: https://www.Insee.Fr/Fr/Statistiques/2012713. Avalaibility of air pollution data: https://Atmo-France.Org/. Avalaibility of health data: https://www.data.gouv.fr/fr/datasets/.
